# Long-term hypoxia modulates depolarization activation of BK_Ca_ currents in fetal sheep middle cerebral arterial myocytes

**DOI:** 10.3389/fphys.2024.1479882

**Published:** 2024-11-05

**Authors:** Nikitha Nelapudi, Madison Boskind, Xiang-Qun Hu, David Mallari, Michelle Chan, Devin Wilson, Monica Romero, Eris Albert-Minckler, Lubo Zhang, Arlin B. Blood, Christopher G. Wilson, Jose Luis Puglisi, Sean M. Wilson

**Affiliations:** ^1^ Lawrence D Longo Center for Perinatal Biology, Loma Linda University School of Medicine, Loma Linda, CA, United States; ^2^ Advanced Imaging and Microscopy Core, Loma Linda University School of Medicine, Loma Linda, CA, United States; ^3^ Department of Biostatistics, California Northstate University School of Medicine, Elk Grove, CA, United States

**Keywords:** smooth muscle, calcium, Ca^2+^ oscillation, Ca^2+^ sparks, high altitude, hypoxia, ion channel

## Abstract

**Introduction:**

Previous evidence indicates that gestational hypoxia disrupts cerebrovascular development, increasing the risk of intracranial hemorrhage and stroke in the newborn. Due to the role of cytosolic Ca^2+^ in regulating vascular smooth muscle (VSM) tone and fetal cerebrovascular blood flow, understanding Ca^2+^ signals can offer insight into the pathophysiological disruptions taking place in hypoxia-related perinatal cerebrovascular disease. This study aimed to determine the extent to which gestational hypoxia disrupts local Ca^2+^ sparks and whole-cell Ca^2+^ signals and coupling with BK_Ca_ channel activity.

**Methods:**

Confocal imaging of cytosolic Ca^2+^ and recording BK_Ca_ currents of fetal sheep middle cerebral arterial (MCA) myocytes was performed. MCAs were isolated from term fetal sheep (∼140 days of gestation) from ewes held at low- (700 m) and high-altitude (3,801 m) hypoxia (LTH) for 100+ days of gestation. Arteries were depolarized with 30 mM KCl (30K), in the presence or absence of 10 μM ryanodine (Ry), to block RyR mediated Ca^2+^ release.

**Results:**

Membrane depolarization increased Ry-sensitive Ca^2+^ spark frequency in normoxic and LTH groups along with BK_Ca_ activity. LTH reduced Ca^2+^ spark and whole-cell Ca^2+^ activity and induced a large leftward shift in the voltage-dependence of BK_Ca_ current activation. The influence of LTH on the spatial and temporal aspects of Ca^2+^ sparks and whole-cell Ca^2+^ responses varied.

**Discussion:**

Overall, LTH attenuates Ca^2+^ signaling while increasing the coupling of Ca^2+^ sparks to BK_Ca_ activity; a process that potentially helps maintain oxygen delivery to the developing brain.

## 1 Introduction

The fetal brain grows rapidly and has high metabolism. Middle cerebral arteries (MCA) are important as they ensure the developing brain receives adequate blood flow and appropriate nutrient and oxygen delivery. These arteries branch from the internal carotid artery from the circle of Willis and have distinctive physiology and pathophysiology relative to arteries in adults. They are uniquely positioned to properly deliver oxygen and nutrients, responding to both acute and chronic changes in the physiological needs of the developing brain.

Fetal MCA are well adapted to meet the metabolic demands of the developing brain. Critically, the systemic vascular pressures of the fetus are far lower than what is found in the newborn, which are lower still than what is found in the adult (Ducsay et al., 2018). While MCAs are designed to protect against hemorrhage, the low perfusion pressures in the fetus cause the arteries to be relatively dilated so that oxygen and nutrient delivery are optimized. The MCA, along with other cerebral arteries, contributes to maintaining proper resistance. Blood flow through the MCA is partially regulated by autoregulatory mechanisms that govern vessel contractility and diameter, ensuring that oxygen and nutrients are delivered adequately to the brain under varying conditions.

Vasoregulation of cerebral arteries is largely controlled by changes in the membrane potential and cytosolic Ca^2+^ concentration. Membrane depolarization activates voltage dependent ion channels that result in whole-cell increases in cytosolic Ca^2+^ arising from a combination of intracellular Ca^2+^ release and extracellular Ca^2+^ entry (Tykocki et al., 2017; Jackson, 2021). The elevation in cytosolic Ca^2+^ causes arterial myocytes to contract, which results in a narrowing of arterial diameter and restriction in brain blood flow. Depolarization of the plasma membrane also activates a negative feedback modulatory pathway, which can prevent hyper constriction and tissue ischemia. This negative feedback pathway results from stimulation of ryanodine receptors (RyR) on the sarcoplasmic reticulum mediated by a sequence of events that includes membrane depolarization and activation of L- and T-type voltage dependent Ca^2+^ channels, collectively termed Ca_v_ (Harraz et al., 2015; Hashad et al., 2017). The plasma membrane Ca^2+^ influx through these voltage gated channels activates RyRs and induces spatially and temporally restricted Ca^2+^ events in sub-sarcolemma regions, often referred to as Ca^2+^ sparks (Jaggar et al., 2000). These Ca^2+^ spark events transiently activate large conductance potassium (BK_Ca_) channels at the plasma membrane, which induces membrane hyperpolarization. The membrane hyperpolarization, in turn, reduces activation of the voltage-activated Ca^2+^ channels and associated Ca^2+^ influx leading to vasorelaxation (Gebremedhin et al., 1994; Brenner et al., 2000; Ledoux et al., 2006; Thorpe et al., 2017).

When considering the influence of hypoxic insult, acute hypoxia, even in the fetus, elicits a brain sparing effect where brain blood flow increases, preserving oxygenation and nutrient delivery (Ashwal et al., 1984). The increases in brain blood flow are due to cerebral arterial dilation, which is due, in part, to augmented activation of the Ca_v_ -RyR- BK_Ca_ signaling axis. Long-term intrauterine hypoxia, in comparison, elicits a variety of adaptations in the fetal cerebral vasculature. Previous evidence from our group illustrates there is a sparing effect that maintains brain oxygenation in a high-altitude intrauterine long-term hypoxic fetal sheep model that includes an increase in fetal hematocrit (Kamitomo et al., 1992; 1993; Ducsay et al., 2018). There are also complex changes in vasodilatory capacity including suppression in Ca^2+^ regulatory process, downregulation of BK_Ca_ channel subunit expression, and increased BK_Ca_ channel affinity for Ca^2+^ (Tao et al., 2015; Thorpe et al., 2017; Ducsay et al., 2018; Reid et al., 2021). These *in utero* adaptations to long-term hypoxia, while beneficial to the fetus, may not allow the animal to thrive after birth as these adaptations may likely impair the ability of the MCA to properly regulate brain blood flow in the newborn.

The current studies were designed to probe the effects of long-term hypoxia on cytosolic Ca^2+^ signals and BK_Ca_ channel activity in middle cerebral arterial myocytes in fetal sheep given previous evidence of underlying dysregulation of MCA vasoreactivity and BK_Ca_ channel expression and function in cerebral arterial myocytes. Previous evidence led us to hypothesize that long-term hypoxia due to high altitude gestation would enhance BK_Ca_ channel activity without much if any change in local or global Ca^2+^ signals (Tao et al., 2015; Thorpe et al., 2017; Reid et al., 2021). Ca^2+^ signals and BK_Ca_ channel activity were studied by examining local and global Ca^2+^ signals and whole-cell K^+^ currents in middle cerebral arterial myocytes of near-term fetal sheep that gestated at low- or high-altitude.

## 2 Materials and methods

### 2.1 Experimental animals

The methods used for the studies in this manuscript were adapted from (Hashad et al., 2017; Silpanisong et al., 2017; Thorpe et al., 2017; Reid et al., 2021) and are based on experimental animal, tissue preparation, confocal imaging, and electrophysiological studies as outlined in (Giang et al., 2016; Hashad et al., 2017; Shen et al., 2018; Hu et al., 2019; 2020; Leslie et al., 2021; Reid et al., 2021). In brief, studies were conducted in accordance with the Animal Welfare Act, the National Institutes of Health *Guide for the Care and Use of Laboratory Animals (* https://grants.nih.gov/grants/olaw/Guide-for-the-Care-and-use-of-laboratory-animals.pdf), “The Guiding Principles in the Care and Use of Animals” approved by the Council of the American Physiological Society and were pre-approved by the Institutional Animal Care and Use Committee of Loma Linda University (IACUC-LLU).

As per our previous studies (Tao et al., 2015; Thorpe et al., 2017; Reid et al., 2021) pregnant ewes of a mixed Western breed were divided into low-altitude (normoxic) and high-altitude long-term hypoxic (LTH) groups. All ewes were obtained from Nebeker Ranch in Lancaster, CA at an elevation near sea level, 720 m. Normoxic control pregnant ewes were maintained near sea level at 720 m for the duration of their gestation. Animals for the LTH groups were held at Nebeker Ranch under normoxic conditions until 30 days gestation at which time the pregnant ewes were transported to the Barcroft Laboratory, White Mountain Research Station in Bishop, CA at an elevation of 3,801 m. Previous work shows that residing at the Barcroft laboratory results in a maternal arterial PO_2_ of 60 ± 3 Torr and a fetal arterial PO_2_ of 19 ± 2 Torr (Kamitomo et al., 1993). Animals remained at elevation for the remaining ∼110 days of gestation for pregnant ewes. Following this acclimatization period, ewes were transported (∼6 h drive) to Loma Linda University (LLU) for study at an elevation of 346 m. Once at LLU, LTH ewes were surgically instrumented with arterial and tracheal catheters. Based on frequent arterial blood gas sampling and adjustment of the rate of N_2_ flow through the tracheal catheter, arterial PO_2_ levels in adult sheep were maintained at ∼60 Torr for 2–4 days, mimicking the high altitude conditions until the day of study (Kamitomo et al., 1993). On the day of experimental study, ewes were induced with thiopental sodium (10 mg/kg iv), and then intubated. Anesthesia of the ewe was maintained via inhalation of 2%–3% isoflurane in O_2_ for the duration of the surgery. Fetuses were delivered via hysterectomy. Generally, the male to female birth ratio is ∼1:1, however for the current studies only 2 female fetuses were identified in each group, precluding any substantive gender based analyses. Following delivery, fetal sheep were euthanized with an overdose of Euthasol (pentobarbital sodium, 100 mg/kg) and phenytoin sodium (10 mg/kg). Middle cerebral arteries were isolated from a total of 6 normoxic and 6 hypoxic fetuses.

### 2.2 Middle cerebral artery isolation

Near term fetal brains of mixed sex were removed and placed in iced Balanced Salt Solution (BSS) of the following composition (mM): 126 NaCl; 5 KCl; 10 HEPES; 1 MgCl_2_; 2 CaCl_2_; 10 glucose; pH 7.4 (adjusted with NaOH). Middle cerebral arteries then were quickly dissected under normoxic conditions in BSS and stored in ice-cold BSS until used for experimental study.

### 2.3 Confocal microscopy studies

Intracellular Ca^2+^ of middle cerebral arterial myocytes was measured *in situ* with a Ca^2+^ sensitive fluorescent dye (Fluo-4 AM, Cat No F14201, Invitrogen, Carlsbad, CA) using a Zeiss 710 NLO laser scanning confocal imaging workstation (Thornwood, NY) with an inverted microscope (Zeiss Axio Observer), using procedures based on previous studies (Harraz et al., 2014; 2015; Hashad et al., 2017; Reid et al., 2021). Fluo-4 AM was dissolved in DMSO creating a 1 mM stock solution. Arteries were placed in BSS containing 10 µM Fluo-4 with 0.1% Pluronic F-127 Cat No P6867 (Invitrogen) from a 20% w/v stock solution in DMSO for 1 h in the dark at room temperature (∼22–24 C). These arterial segments were subsequently washed with BSS for 30 min to facilitate dye esterification and were then cut into linear strips for experimental studies. Arterial myocytes were depolarized by treating arteries with a solution where there was an equimolar replacement of 30 mM NaCl for 30 mM KCl (30K) as we have performed previously (Hadley et al., 2012; Reid et al., 2021). The resting membrane potential based on examinations of middle cerebral arterial myocytes from cats, dogs, rats, and rabbits is predicted to range from roughly −70 mV to −40 mV (Harder, 1980; Fujiwara et al., 1982; Knot and Nelson, 1995; McPherson and Keily, 1995; Nelson et al., 1995; McNeish et al., 2010). Based on the Nernst reversal potential for potassium and a predicted intracellular potassium concentration of ∼ 140 mM (Wilson and Leblanc, 2005), the membrane is expected to depolarize to −40 mV; which we have previously shown to increase Ca^2+^ spark activity in basilar and pulmonary arterial myocytes (Reid et al., 2021; Hadley et al., 2012) and to elicit pulmonary arterial contractility (Papamatheakis et al., 2012). Arteries were then treated with 10 µM ryanodine in the presence of 30K for 30 min prior to imaging to reduce RyR activity (Janiak et al., 2001; Hadley et al., 2012).

Imaging was performed in arterial strips that were pinned to a Sylgard block in an *en face* orientation (Ellsworth Adhesives, Germantown, WI) with fine insect dissecting pins and placed into an open bath imaging chamber (Warner Instruments, Hamden, CT). The Fluo-4 was illuminated at 488 nm via a Krypton-argon laser. Emitted light was captured with a photomultiplier tube with a band limited range of 493–622 nm in both full frame and line scan imaging studies using a Zeiss Apochromat 63X Water Immersion 1.2 N.A. objective. All confocal imaging experiments were performed at Loma Linda University (346 m) under normobaric and normoxic conditions at room temperature (∼24°C) in the dark.

Time lapse recordings of multiple myocytes and subcellular Ca^2+^ recordings of individual myocytes were made in each arterial preparation as we have performed previously in tissues from multiple species (Hadley et al., 2012; Shen et al., 2018; Reid et al., 2021; Hashad et al., 2017; Hu et al., 2020). Whole-cell Ca^2+^ oscillatory activity of individual cells was assessed in time series recordings of 512 × 512 full frame images. These images were made with a lateral pixel size of 0.264 μm per pixel and an imaging depth adjusted to 5.4 μm, which is roughly the width of an arterial myocyte based on prior morphological studies in live and fixed cells (Hadley et al., 2012; Shen et al., 2018). These recordings ranged from 234 to 391 s at a frame rate of 1.28 Hz, which we have previously used for spatial and temporal examination of spontaneous whole-cell Ca^2+^ oscillations and stimulated responses (Shen et al., 2018; Reid et al., 2021; Leslie et al., 2021). Time series recordings were followed by 30–50 line scan recordings made along the edge of the sarcolemma at the axial center of the cell, which were made to measure subcellular Ca^2+^ spark activity. Each line scan was 18.9 s at a frequency of 529 Hz, with one line being made in each cell. The lateral pixel size for the 1,421 line-scan recordings that were made for these studies was 0.05 ± 0.03 μm per pixel. The pinhole was adjusted to an imaging depth of 2.5 μm, which is about 50% of the width of a myocyte based on prior morphological studies in live cells (Hadley et al., 2012; Shen et al., 2018).

### 2.4 Measurement of BK_Ca_ channel currents

Smooth muscle cells were enzymatically dissociated from fetal lamb middle cerebral arteries. Briefly, arteries were cut into ring segments and incubated (37°C, 10 min) in low-Ca^2+^ HEPES-buffered physiological salt (PSS) solution containing (in mmol/L) 140.0 NaCl, 5.0 KCl, 0.1 CaCl_2_, 1.2 MgCl_2_, 10.0 HEPES, and 10.0 glucose (pH 7.4). Vessels were then exposed to a 2-step digestion process that involved: (1) a 45 min incubation in low-Ca^2+^ HEPES-buffered PSS (37°C) containing 1.5 mg/mL papain (Worthington Biochemical; Lakewood, NJ), 1.5 mg/mL dithiothreitol (Millipore Sigma, Burlington, MA), and 1.5 mg/mL BSA (Millipore Sigma) and (2) a 45 min incubation in low-Ca^2+^ HEPES-buffered PSS (37°C) containing 1.5 mg/mL collagenase IV (Worthington Biochemical), and 1.5 mg/mL BSA (Millipore Sigma). After the enzyme treatment, tissues were washed with low-Ca^2+^ HEPES-buffered PSS. Single smooth muscle cells were released by gently inverting the tube(s) containing low-Ca^2+^ HEPES-buffered PSS and digested tissues several times. The cells were kept at 4°C and experiments were conducted within 6 h of cell isolation.

Spontaneous transient outward currents (STOCs) were recorded in the whole-cell configuration of the perforated patch-clamp technique using an EPC 10 patch-clamp amplifier with Patchmaster software (HEKA, Lambrecht/Pfalz, Germany) at room temperature as previously described (Hu et al., 2019). Briefly, cell suspension drops were placed in a recording chamber, and adherent cells were continuously superfused with HEPES-buffered PSS containing (in mmol/L) 140.0 NaCl, 5.0 KCl, 1.8 CaCl_2_, 1.2 MgCl_2_, 10.0 HEPES, and 10.0 glucose (pH 7.4). Only relaxed and spindle-shaped myocytes were used for recording. Micropipettes were pulled from borosilicate glass and had resistances of ∼ 5 megaohm (mΩ) when filled with a pipette solution containing (in mmol/L) 140.0 KCl, 1.0 MgCl_2_, 5.0 Na_2_ATP, 5.0 EGTA, and 10.0 HEPES (pH 7.2) with 250 μg/mL amphotericin B. Membrane currents were recorded while the cells were held at steady membrane potentials between −50 and 10 mV, which were increased in 10 mV increments of 1 min duration. STOCs were analyzed with Mini Analysis Program (Synaptosoft, Leonia, NJ) with a threshold for detection set at 10 pA. The currents were normalized to cell capacitance and expressed as picoampere per picofarad (pA/pF). Inhibitory effects of iberiotoxin and tetraethylammonium were examined in fetal hypoxic middle cerebral arterial myocytes at a holding potential of +10 mV. Because of inherent low STOC activity in myocytes isolated from normoxic animals these studies were only performed in hypoxic animals.

### 2.5 Line scan analysis

Ca^2+^ sparks recorded in cerebral arterial myocytes were analyzed using an automated approach with Sparklab 5.8 as recently described (Boskind et al., 2023). The analysis identified Ca^2+^ spark events and characterized Ca^2+^ spark morphology and spatial-temporal features of each event. Preliminary analysis worked towards the goal of optimizing the threshold used for Ca^2+^ spark detection and generating critical cutoffs for automated analysis. An evaluation of the threshold for Ca^2+^ spark responses above background noise was performed using various thresholds for spark detection including 0.5, 0.6, 0.7, 1.0, and 1.5 above background noise. A total of 214 true positive Ca^2+^ spark events were manually observed in 19 data traces imaged in myocytes of arterial segments isolated from 4 hypoxic fetuses, and comparisons were made to automated detection at various thresholds. From these data, the number of true positives, false positives, and false negatives were determined. This allowed for the calculation of the positive predictive value (PPV), sensitivity, and false discovery rate (FDR). The resultant analysis of Ca^2+^ spark events was made at a threshold of 0.7 as it provided a balance between the sensitivity of spark detection and the positive predictive value, which is the same cutoff we used previously (Boskind et al., 2023). Analysis was performed as we have done previously (Reid et al., 2021; Shen et al., 2018; Hu et al., 2019), and included the number of cells present with Ca^2+^ sparks, the frequency of activation as well as the event amplitude, full width at half maximum (FWHM), full duration at half maximum (FDHM), and the time constant for decay (Tau). These automatically detected features of the events were then filtered using an interquartile range (IQR) fencing method for amplitude (Lower and Upper), FWHM (Lower and upper), FDHM (Lower and Upper), and Tau (Lower and Upper), and statistical comparisons made (Boskind et al., 2023). The outlier fencing methods were based on the first and third quartiles (Q1, Q3) and the IQR. The lower inner fence was Q1 – (n × IQR), while the upper outer fence was Q3 + (n × IQR), where n represents 1.5. Percentiles, quartiles, and interquartile ranges were obtained via Prism 10.2.2. The total number of events examined along with the numbers examined after filtration with statistical fencing are provided in Table 1.

**TABLE 1:** Number of Ca^2+^ spark observations before and after data fencing.

	Normoxic			Hypoxic		
	Control	30K	30K+Ry	Control	30K	30K+Ry
Total	1,632	8,106	149	1,693	4,943	469
IQR 1.5 Number of Events						
Amplitude	1,544	7,963	143	1,619	4,790	441
FWHM	1,378	6,620	69	1,262	4,037	410
FDHM	1,590	7,966	148	1,644	4,869	460
Tau	1,516	7,538	133	1,391	4,578	386

A total of 16,992 events were evaluated across all arterial segments, animals, and conditions. Recordings were made in myocytes of arteries isolated from 6 normoxic and 6 hypoxic fetal sheep. 240 line scans were made for each condition in normoxic animals. In hypoxic animals, 241 control, 240 30K, and 220 30K + 10 μM ryanodine (Ry) line scan recordings were made. Data are presented in Figures 2–5.

### 2.6 Ca^2+^ oscillatory signal analysis

As described in some detail, regions of interest with Ca^2+^ oscillations were detected automatically *post hoc* using the LCPro plug-in for ImageJ (Shen et al., 2018; Reid et al., 2021). For presentation purposes, the fractional fluorescence intensity was automatically calculated using LCPro (Shen et al., 2018) following image registration and image cropping to a final size of 490 × 490 pixels. Regions of interest were detected for changes in fractional fluorescence in 1.56 μm (six pixel) defined regions, with the region size determined empirically as it allows for examination of fluorescence changes in individual cells as opposed to larger regions, which often overlap adjacent cells (Shen et al., 2018; Reid et al., 2021). For the purposes of the current study, erroneous data was removed using analytical techniques recently described (Boskind et al., 2023). This was achieved by using an outlier fencing approach to create upper and lower critical cutoffs along with an empirical approach of removing events shorter than 6 s or longer than 40 s. Further statistical analysis was performed, as described below. The total number of events examined along with the numbers examined after filtration with statistical fencing are provided in Table 2.

**TABLE 2:** Number of Ca^2+^ oscillation observations before and after data fencing.

	Normoxic			Hypoxic		
	Control	30K	30K+Ry	Control	30K	30K+Ry
Total	787	1,312	129	595	583	93
IQR 1.5 Number of Events						
Amplitude	234	923	85	349	465	63
AUC	294	899	83	351	465	62
Rise Time	398	901	77	333	461	56
Duration	432	900	72	331	457	54

A total of 3,499 events were examined across all arterial segments, animals, and recording conditions. Recordings were made in arteries isolated from 6 normoxic and 6 hypoxic fetal sheep. Data are presented in Figures 10–13.

### 2.7 Spatial - temporal Ca^2+^ signaling analysis

The spatial and temporal characteristics to the whole-cell Ca^2+^ oscillatory events were analyzed using customized processes outlined previously (Shen et al., 2018; Reid et al., 2021). This was performed to provide a better understanding of Ca^2+^ signaling networks within and between myocytes in the arterial wall. In brief, a cross correlation analysis for each Ca^2+^ event was performed in time and space for each region of interest detected by LC Pro (Shen et al., 2018). As per our previous studies, a correlation coefficient of r = 0.8 was used for all data sets (Shen et al., 2018; Reid et al., 2021). “Friends” were determined to be calcium transients that had a correlation coefficient greater than r = 0.8. “Neighbors” were defined as calcium transients occurring in myocytes within a radius of 100 pixels (∼26 µm), which was chosen because most all adjacent cells were within this distance.

### 2.8 Chemicals reagents and drugs

Most reagents and chemicals were purchased from Sigma-Aldrich (St. Louis, MO) and Millipore Sigma (Burlington, MA). Fluo-4 AM and Pluronic F127 were purchased from Invitrogen, a division of Thermo Fisher (Waltham, MA) while iberiotoxin and ryanodine were purchased from Tocris (Ellisville, MO).

### 2.9 Statistical methods and sampling

All time-series recordings were graphed, and statistical analyses performed with Prism 10.2.2. Contingency analysis was used in Figure 3A to examine Ca^2+^ spark activity based on the number of cells with and without events, with statistical differences examined by a chi square test. The data are displayed as the percentage of cells firing with Ca^2+^ sparks for each treatment and group. Histograms shown in Figures 4, 10 are the number of responding events in each bin and were tallied across all experimental treatments and groups. The large number of data replicates are suggestive that the data are derived from a population events. Summarized data for the Ca^2+^ spark events and Ca^2+^ oscillatory signals are presented as individual data points with mean and 95% confidence intervals. Data were evaluated for normality using a D’Agostino-Pearson test prior to any comparative statistical analysis. The specific test used is denoted in the figure legend or table. When the data were normally distributed a two-way repeated measures ANOVA with a Bonferroni’s multiple comparison test was used. When the data were not normally distributed then a Kruskal–Wallis one-way ANOVA with Dunn’s multiple comparison test. P values for ANOVA were adjusted for multiplicity for each comparison. P < 0.05 was considered statistically significant, unless otherwise noted. The P values are provided on the figure and denoted on the figure legend. The number of studies performed are provided as are the number of events, arteries, animals, or scans examined. The exact details are provided along with each figure and in Tables 1, 2.

## 3 Results

**FIGURE 1:**
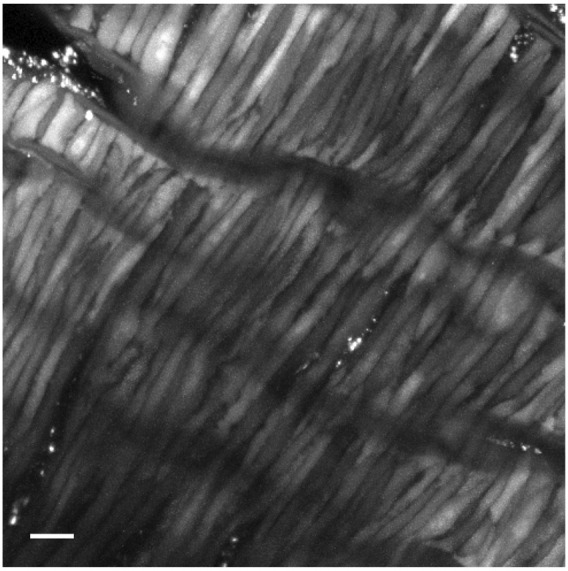
Representative image of a Fluo-4 loaded middle cerebral arterial segment from a fetal normoxic sheep recorded *en face* in the presence of 30 mM K^+^. Shown is the grayscale maximum intensity projection from 476 images recorded over 371 s. Scale bar is 10 microns. Recording was made with a 1.2 NA ×63 water immersion objective. Image brightness and contrast were adjusted to improve visualization of cells.

**FIGURE 2:**
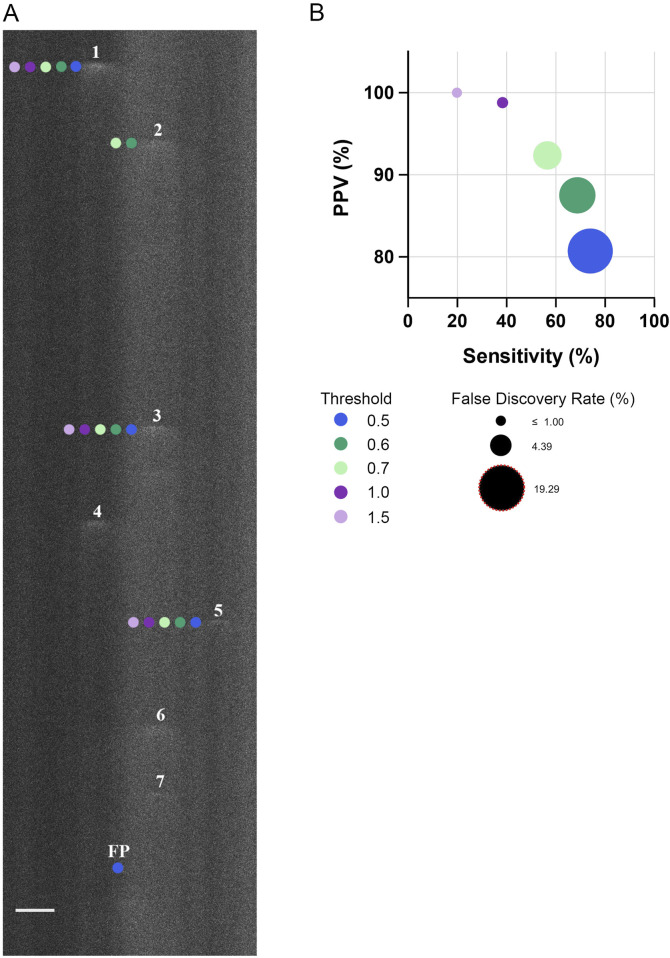
Threshold for identifying Ca^2+^ spark events in line-scan recordings impacts the sensitivity, positive predictive value, and false discovery rate. **(A)** Representative fluo-4 fluorescence line scan recording in a single fetal cerebral arterial myocyte. Recording was performed in a myocyte from an artery of a hypoxic fetus recorded *en face* under control conditions. Numbers indicate Ca^2+^ sparks identified by a trained observer whereas neighboring-colored dots indicate the Ca^2+^ sparks identified at various thresholds by Sparklab 5.8 as denoted in the adjoining legend. **(B)** The sensitivity of identifying a Ca^2+^ spark as a function of the positive predictive value (PPV) along with the false discovery rate (FDR) at various thresholds. Scale Bar is 5 microns. A total of 214 Ca^2+^ spark events from 19 line scan recordings from 2 hypoxic animals recorded under control conditions were made to determine the sensitivity, PPV and FDR. Line scan recording was made with a 1.2 NA ×63 water immersion objective at 529 Hz. Image brightness and contrast were adjusted to improve visualization of cells.

Figure 1 shows a full frame maximum intensity projection from a time series of Fluo-4 loaded middle cerebral arterial myocytes from a fetal normoxic sheep that was used for analysis of Ca^2+^ oscillatory activity. This depiction highlights the spindle-shaped morphology of the myocytes, which are closely associated with one another, a characteristic consistent with observations in various vascular beds and species (Hadley et al., 2012; Harraz et al., 2014; 2015; Shen et al., 2018; Hu et al., 2020; Reid et al., 2021). Punctate staining is also observed, which is indicative of dye bound to the adventitia, or in damaged/overloaded myocytes. Line-scan images, such as that depicted in Figure 2A were made on the inside edge of the sarcolemma, which is where we and others have previously shown that Ca^2+^ sparks occur (Nelson et al., 1995; Jaggar et al., 2000; Janiak et al., 2001).

### 3.1 Calcium spark activity and morphologic variability

The first series of studies were performed to examine Ca^2+^ spark activity in arterial myocytes. Preliminary analyses involved a determination of the optimal threshold for analysis of our dataset through a comparison of the spark detection capabilities of Sparklab 5.8 at multiple thresholds, using approaches based on our recent work (Boskind et al., 2023). Figure 2A showcases this comparative process in a representative line-scan image overlaid with markings denoting the individual Ca^2+^ events automatically detected at various thresholds by SparkLab and those that were manually detected by a trained observer. The image demonstrates how Ca^2+^ sparks with lower fluorescence intensities are increasingly indistinguishable from surrounding background noise at higher fluorescence thresholds, resulting in a decrease in the sensitivity of event detection. In comparison, at lower thresholds there was an elevation in the false positive rate as slight increases in fluorescence are more likely to be incorrectly recognized as a Ca^2+^ spark by SparkLab. To quantify the effectiveness and accuracy of each threshold to detect Ca^2+^ sparks, the sensitivity, positive predictive value (PPV), and false discovery rate (FDR) were calculated and graphed in Figure 2B. Notably, even at the least rigorous threshold of 0.5, the program detected Ca^2+^ sparks with a PPV and FDR of 80% and 19%, respectively. The FDR decreased to 12% and 7% percent at 0.6 and 0.7 threshold, respectively. Sensitivity incrementally decreased with increasing threshold through the removal of lower-quality events that were not substantially out of the noise floor. Based on this threshold analysis, we empirically proceeded with the analysis of our dataset at the 0.7 threshold as this criterion allowed for a balance between detecting “true” Ca^2+^ sparks while limiting the number of false positives. As we have recently shown, modifying the threshold has minimal or no impact on the spatial and temporal aspects to Ca^2+^ sparks (Boskind et al., 2023).

**FIGURE 3:**
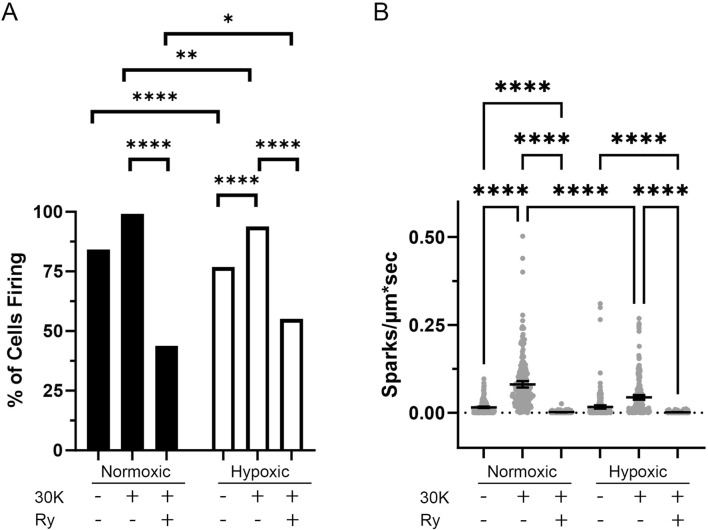
Long-term hypoxia reduces the activity of Ca^2+^ sparks in middle cerebral arterial myocytes of fetal sheep. **(A)** Percentage of line scans with detectable Ca^2+^ sparks. **(B)** Ca^2+^ spark firing frequency in each line scan. Ca^2+^ sparks of myocytes were recorded using line scan approaches in the absence (control) and presence of 30 mM K^+^ (30K) or 30K with 10 μM ryanodine (Ry). Bars represent mean ± 95% CI. Data were analyzed either with a chi square test **(A)** or with a Kruskal–Wallis one-way ANOVA with Dunn’s multiple comparison test **(B)**. **p < 0.01, ***p < 0.001 ****p < 0.0001. For normoxic animals, 240 line scans were performed under each recording condition. For hypoxic animals, a total of 241 control, 240 30K and 220 30K + 10 μM ryanodine line scan recordings were made.

Delving into the central aim of our study, we explored the influence of long-term hypoxia on Ca^2+^ spark events in fetal sheep middle cerebral arterial (MCA) myocytes. Even under control conditions Ca^2+^ sparks occurred in 84% of the normoxic and 77% of the hypoxic myocytes (Figure 3A). Membrane depolarization with 30K increased the proportion of cells with Ca^2+^ sparks (Figure 3A) and their frequency of activation (Figure 3B), while the ryanodine treatment decreased the number of cells with Ca^2+^ sparks and their frequency in both normoxic and hypoxic groups, illustrating that these are classic Ca^2+^ sparks due to activation of ryanodine receptors (Jaggar et al., 2000; Janiak et al., 2001; Hadley et al., 2012). Long-term hypoxia, however, reduced the proportion of myocytes with Ca^2+^ spark activity relative to normoxia in control and 30 mM K^+^ (30K) groups while the frequency of Ca^2+^ spark activity was attenuated by long-term hypoxia in the 30K group.

**FIGURE 4:**
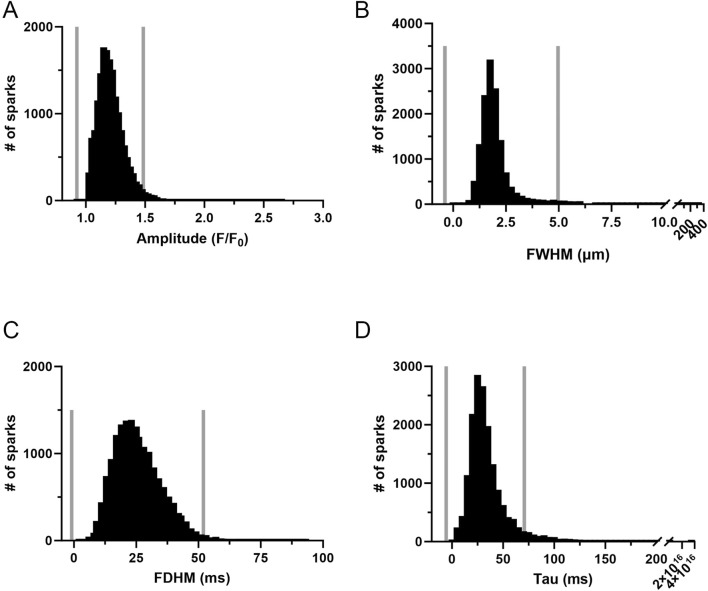
Frequency distribution and data filtering methods for spatial and temporal aspects of Ca^2+^ sparks in middle cerebral arterial myocytes of fetal sheep. Histogram plots of Ca^2+^ spark **(A)** amplitude, **(B)** full width at half maximum (FWHM), **(C)** full duration at half maximum (FDHM), and **(D)** Tau. Values are numbers of events with responses within each bin. Gray vertical lines provide upper and lower IQR 1.5 limits. Responses were compiled across all 12 animals and recording conditions, this being a total of 16,992 events made in 1,421-line scan recordings from middle cerebral arterial segments of 6 normoxic and 6 hypoxic animals.

The influence of long-term hypoxia on the spatial and temporal aspects to the Ca^2+^ sparks were then examined. Figure 4 shows histogram plots for the various morphological spark parameters that were examined including the amplitude, full-width at half maximum (FWHM), full-duration at half maximum (FDHM), and tau, where events were compiled across all groups and experimental conditions. Employing outlier fencing techniques, as outlined in our recent work (Boskind et al., 2023), we filtered the dataset to include only those events that met specific criteria. This involved applying a threshold based on the interquartile range (IQR) for data across all groups and experimental conditions multiplied by IQR 1.5 for the data depicted in each of the panels.

**FIGURE 5:**
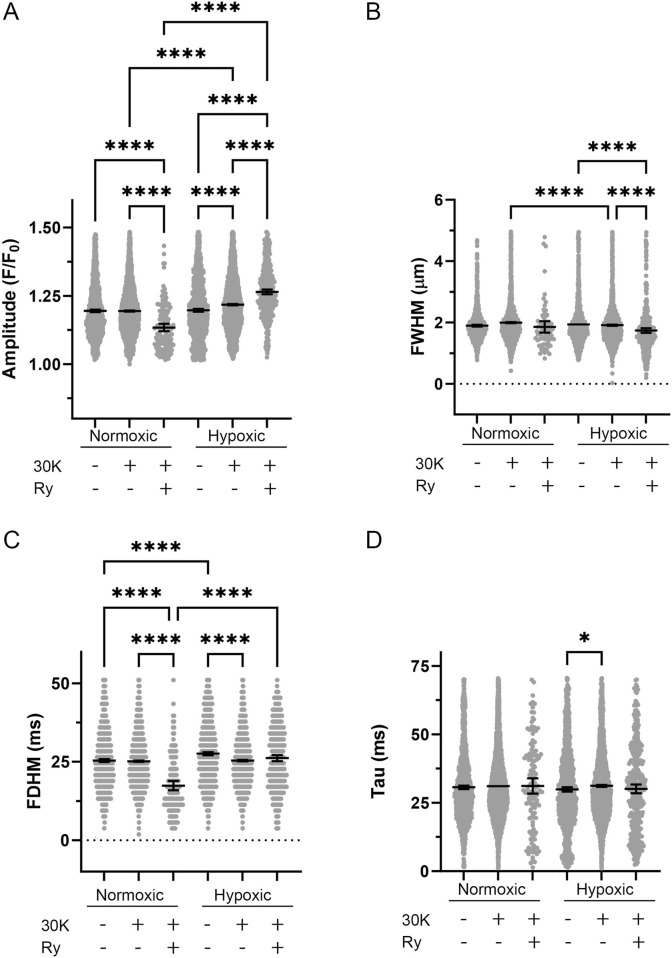
Spatial-temporal aspects to Ca^2+^ sparks were minimally influenced by LTH, membrane depolarization, or ryanodine. **(A)** amplitude, **(B)** full width at half-maximum, **(C)** full duration at half-maximum, and **(D)** tau exposed to control, 30K, or 30K with 10 μM ryanodine (Ry) for Ca^2+^ spark events of arterial myocytes from fetal sheep under normoxic and hypoxic conditions. Bars represent mean ± 95% CI for each parameter. Data were analyzed by a Kruskal–Wallis one-way ANOVA with Dunn’s multiple comparison test based on ranks for each group *P < 0.05, ****P < 0.0001. Responses were examined in 1,421-line scan recordings from middle cerebral arterial segments of 6 normoxic and 6 hypoxic animals. N values for each parameter, condition, and group are provided in Table 1.

Once outliers were removed the various morphological parameters were measured and compared by experimental condition and for the effect of long-term hypoxia. As shown in Figure 5, Ca^2+^ spark events in myocytes of fetal myocytes were modified by the treatment and by long-term hypoxia. Figure 5A illustrates that in myocytes from normoxic animals, ryanodine treatment in the presence of 30K reduced Ca^2+^ spark amplitude. In myocytes from hypoxic animals, 30K with or without ryanodine increased Ca^2+^ spark amplitude. Relative to Ca^2+^ sparks from normoxic myocytes, 30K treatment also increased Ca^2+^ spark amplitude in myocytes from hypoxic animals. Figure 5B shows that Ca^2+^ spark width was less impacted by the treatments and by LTH. Ryanodine in the presence of 30K caused Ca^2+^ sparks to be narrower in myocytes from normoxic animals. Similarly, following 30K treatment Ca^2+^ sparks were narrower in myocytes from hypoxic relative to normoxic animals. Figure 5C shows that the duration of Ca^2+^ sparks was not systematically affected by the treatments or exposure to hypoxia. 30K preferentially decreased the duration of Ca^2+^ sparks in hypoxic animals while ryanodine in the presence of 30K shortened Ca^2+^ spark duration in normoxic myocytes. Under control conditions and following treatment with 30K and ryanodine, Ca^2+^ sparks in myocytes from LTH exposed fetuses had longer durations as compared to myocytes from normoxic animals. Still, Figure 5D shows that the tau for the decay of the Ca^2+^ signal was not affected in the same manner as the duration. The only difference in the time constant for decay of the Ca^2+^ signal was that the tau was increased in the 30K condition relative to control in myocytes from hypoxic animals.

### 3.2 Spontaneous transient outward currents

**FIGURE 6:**
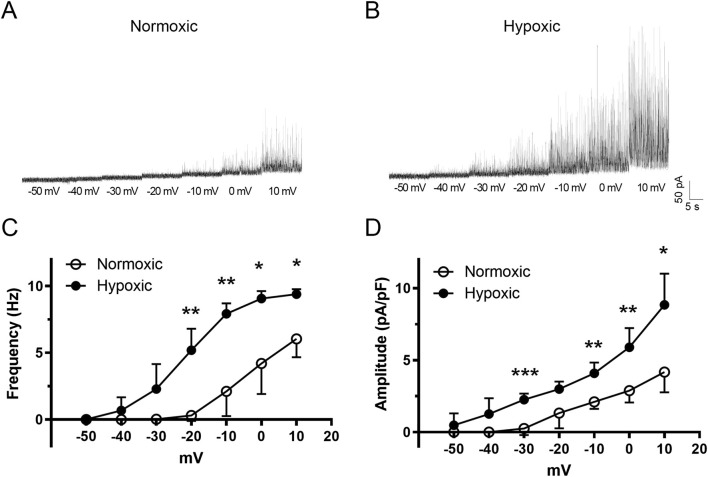
The voltage dependence of activation for spontaneous transient outward currents (STOC) in fetal middle cerebral arterial myocytes experiences a leftward shift following LTH. Raw tracings of STOC activity at various membrane potentials for myocytes isolated from MCA of **(A)** normoxic and **(B)** hypoxic fetal sheep. **(C)** frequency and **(D)** amplitude of STOC activity at various membrane potentials. Bars represent mean ± 95% CI for STOC frequency or amplitude at each membrane voltage. Data were analyzed by a two-way repeated measures ANOVA with a Bonferroni’s multiple comparison test for each group *P < 0.05, **P < 0.01, ***P < 0.001. Recordings were made in myocytes isolated from 5 normoxic, and 6 hypoxic fetuses.

We next examined the spontaneous activity of BK_Ca_ channels using patch-voltage clamp because Ca^2+^ sparks drive BK_Ca_ activity (Harraz et al., 2015; Hashad et al., 2018) and this axis is critical to feedback regulation of vascular tone (Jaggar et al., 2000; Ledoux et al., 2006; Jackson, 2021). Spontaneous outward currents are shown for middle cerebral arterial myocytes of fetal normoxic (Figure 6A) and hypoxic (Figure 6B) sheep for membrane voltages of −50 mV to +10 mV. As expected, the frequency of STOC activity increased with membrane depolarization in myocytes from both normoxic and hypoxic animals. The frequency of activity, however, was increased at more negative membrane potentials in the myocyte from the LTH animal. Figure 6C shows that the frequency of STOC activity was significantly increased by LTH. In myocytes from LTH sheep, the voltage dependence for STOC activation was shifted leftward along the voltage axis, such that there was greater channel activity at more negative membrane potentials. Figure 6D shows that STOC amplitude was also significantly greater at multiple membrane voltages following LTH.

**FIGURE 7:**
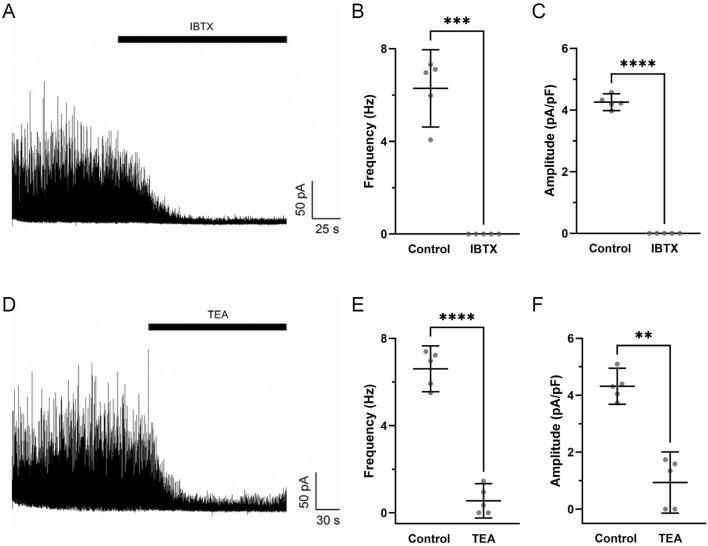
Iberiotoxin and tetraethylammonium inhibit spontaneous transient outward currents in fetal hypoxic middle cerebral arterial myocytes. Raw tracings of STOC activity at a membrane potential of +10 mV in the absence and presence of **(A)** 100 nM IBTX or **(D)** 1 mM TEA in myocytes isolated from MCA of hypoxic fetal sheep. The **(B,E)** frequency and **(C,F)** amplitude of STOC activity in the absence or presence of IBTX or TEA. Bars represent mean ± 95% CI for each parameter. Data were analyzed by a paired t-test in the absence and presence of the channel blockers for each group **P < 0.01, **P < 0.001, ****P< 0.0001. Recordings were made in myocytes isolated from hypoxic fetuses in the presence and absence of IBTX (N = 5) and TEA (N = 5).

Pharmacological studies were then performed to assess whether STOC activity was due to the activation of BK_Ca_ channels (Hu et al., 2020). Figure 7 shows that STOC frequency and amplitude were both reduced by either 100 nM iberiotoxin (Figures 7A–C) or 1 mM Tetraethylammonium (TEA) (Figures 7D–F).

### 3.3 Calcium oscillatory signals and morphological variability

**FIGURE 8:**
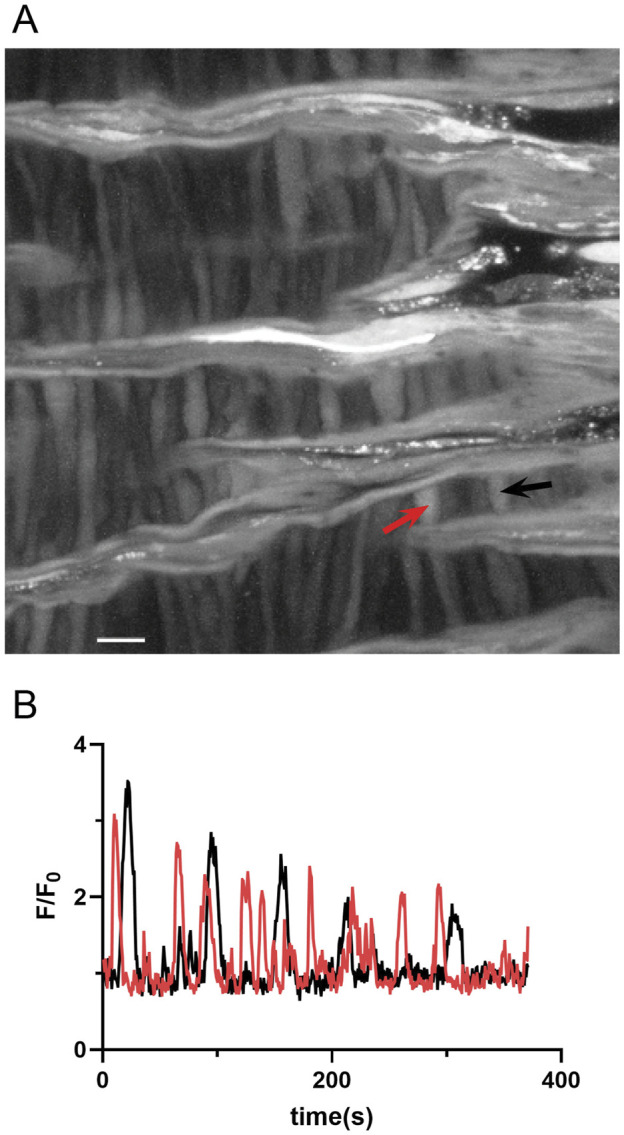
Representative spontaneous whole-cell Ca^2+^ oscillatoions in middle cerebral arterial myocytes from a fetal normoxic sheep recorded *en face* in the presence of 30 mM K^+^. **(A)** Maximum intensity projection for Fluo-4 fluorescence of recorded cells using laser scanning confocal microscopy. Red and black arrows point to regions of interest in two individual myocytes for **(B)** fluorescence intensity tracing showing spontaneous Ca^2+^ oscillations in the two ROIs denoted by the arrows. Scale bar is 10 microns. Recording was made with a 1.2 NA ×63 water immersion objective. Image brightness and contrast were adjusted to improve visualization of cells.

Our investigation then aimed to assess the impact of membrane depolarization and long-term hypoxia (LTH) on Ca^2+^ oscillatory activity in middle cerebral arterial myocytes of fetal sheep. Figure 8A shows a maximum intensity projection of Fluo-4 fluorescence in myocytes of the middle cerebral arterial wall obtained from a time series recording. Figure 8B displays the Fluo-4 fluorescence over time recorded from two regions of interest (ROI’s) in individual myocytes, automatically detected and analyzed using LCPro, a custom analysis program (Francis et al., 2012; Shen et al., 2018). These recorded events, previously categorized as “medium duration,” were prevalent in whole-cell Ca^2+^ recordings, characterized by oscillations spreading uniformly through the myocytes and were empirically determined to last between 6 and 40 s (Reid et al., 2021). Shorter duration events appeared to be in discrete subcellular regions and indicative of Ca^2+^ spark events as previously described (Reid et al., 2021).

**FIGURE 9:**
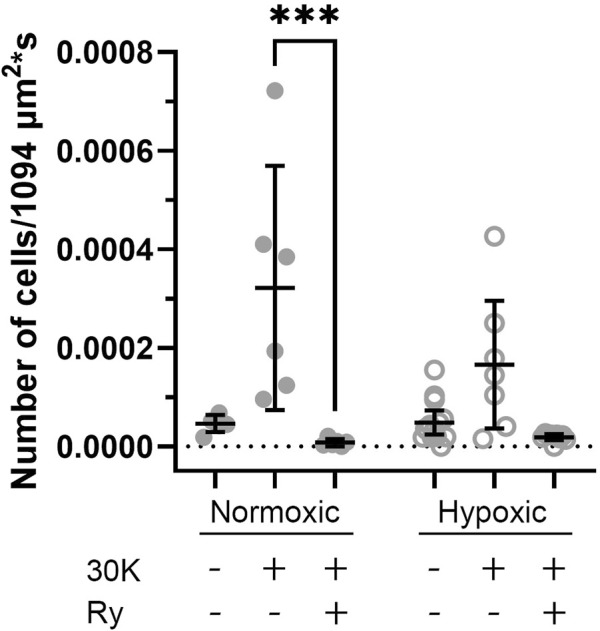
Long-term hypoxia has little impact on the percentage of middle cerebral arterial myocytes with Ca^2+^ oscillations in fetal sheep. Each dot represents the firing rate of cells with Ca^2+^ oscillations under control or with treatment of 30K in the presence or absence of 10 μM ryanodine (Ry) based on an examination of myocytes in 1,094 μm^2^ regions of interest. Replicates were performed in 3 separate regions per recording for fetal normoxic (6 animals with 6 control, 6 30K, and 6 30K + ryanodine recordings), fetal hypoxic (6 animals with 14 control, 7 30K, and 9 30K + ryanodine recordings). ***P < 0.001, ****P< 0.0001 indicates significance based on a Kruskal–Wallis one-way ANOVA with Dunn’s multiple comparison.

To better understand the holistic impact of LTH on Ca^2+^ oscillatory events we examined the number of events that occurred in sampled regions for each time series recording. These data are presented in Figure 9, where the total number of events in each video is presented. There was significant variability in the activity between the various recordings, which we believe limited our ability to determine if there are significant changes in cell activity due to hypoxia, membrane depolarization, or with ryanodine treatment. The only significant difference worth noting was that ryanodine in the presence of 30K reduced the number of events in normoxic MCA relative to membrane depolarization with 30K.

**FIGURE 10:**
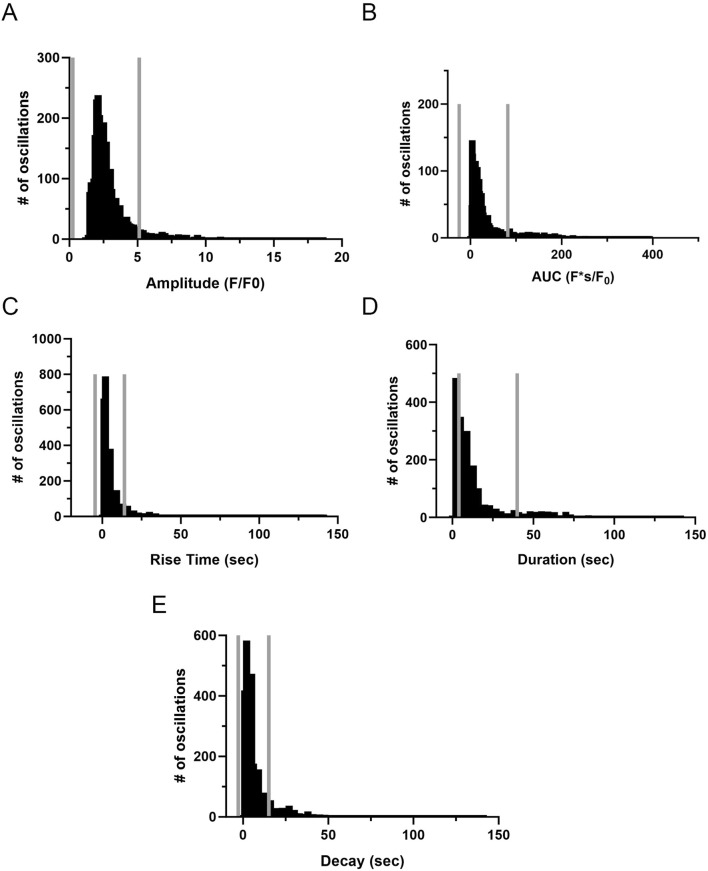
Frequency distribution and data filtering methods for spatial and temporal aspects of Ca^2+^ oscillations recorded from regions of interest in middle cerebral arterial myocytes of fetal sheep. Histogram plots of calcium oscillation **(A)** amplitude, **(B)** area under the curve (AUC), **(C)** rise time, **(D)** duration, and **(E)** decay. Values are numbers of oscillatory events within each bin. Gray vertical lines along the abscissa provide upper and lower IQR 1.5 limits for each examined parameter. Responses were compiled across all animals and conditions; this being obtained from 3,499 oscillatory events from recordings made in myocytes of arterial segments of 6 normoxic and 6 hypoxic animals.

The investigation into Ca^2+^ oscillations encompassed an examination of the event amplitude, area under the curve (AUC), event duration, rise time, and decay. Figures 10A–E presents the graphical representation of events from distinct ROIs. We again performed outlier fencing techniques using IQR 1.5, for data across all groups and experimental conditions, with data outside the gray fences being removed from analysis. The duration of the oscillatory events was evaluated over the range of 6–40 s as opposed to using the IQR 1.5 values. This was done based on empirical knowledge of the duration of Ca^2+^ oscillatory signals, where we previously identified short duration events as Ca^2+^ sparks while long-duration Ca^2+^ events displayed uniquely distinct properties (Shen et al., 2018; Reid et al., 2021).

**FIGURE 11:**
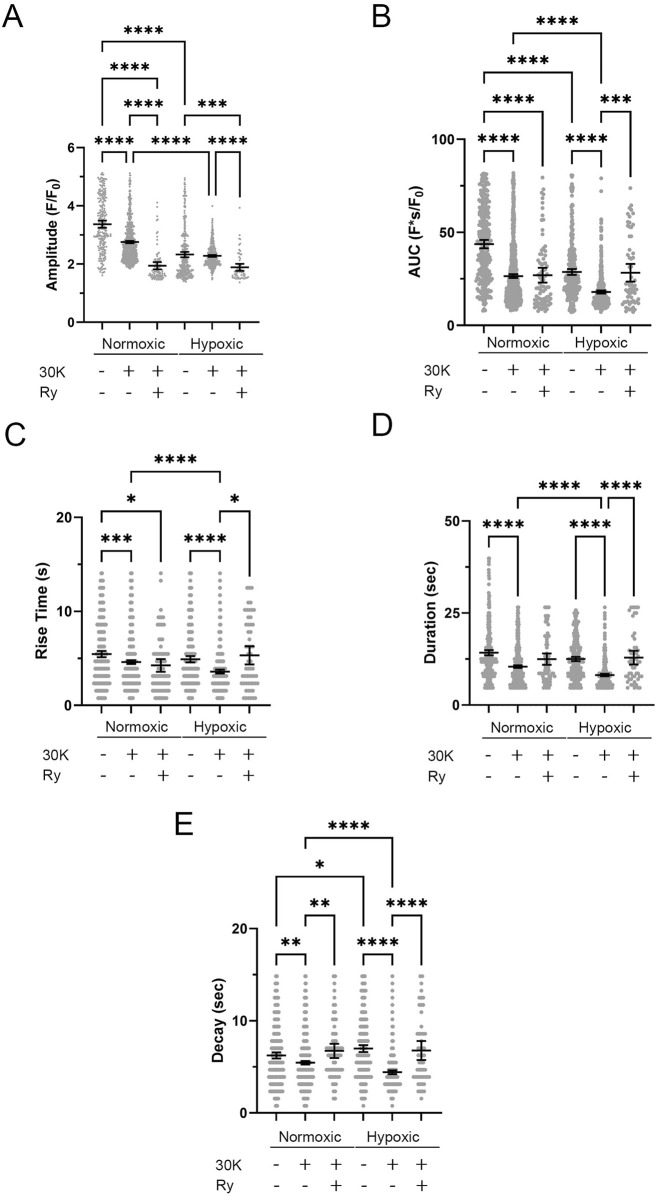
Long-term hypoxia reduces Ca^2+^ oscillations in middle cerebral arterial myocytes of fetal sheep. Effects of membrane depolarization with 30K in the presence and absence of ryanodine (Ry) and long-term hypoxia on **(A)** amplitude of the fractional fluorescence, **(B)** area under the curve, **(C)** rise time, **(D)** duration, and **(E)** decay of the event. Bars represent mean ± 95% CI for each parameter; circles specify individual responses in each condition. *P < 0.05, ***P < 0.001, ****P < 0.0001 indicates significance based on a Kruskal–Wallis ANOVA with a Dunn’s multiple comparisons test based on ranks. The various oscillatory responses were measured in recordings made in myocytes of arterial segments of 6 normoxic and 6 hypoxic animals. Numbers of observations for each parameter, recording condition, and group are provided in Table 2.

Quantitative analysis of the spatial and temporal aspects to the Ca^2+^ oscillatory events revealed nuanced effects of the treatments and experimental groups. The amplitude (Figure 11A) of the Ca^2+^ oscillations varied significantly across the treatment groups. The amplitude of the oscillations in the normoxic control group was significantly higher than in all other groups. Treatment with 30K decreased the amplitude of the oscillations as compared to controls. Interestingly, ryanodine treatment in the presence of 30K resulted in a further reduction in oscillatory amplitude. The area under the Ca^2+^ oscillation curve (Figure 11B) was modified by long-term hypoxia and by the treatment condition. Oscillations in the normoxic control group had the largest AUC, with oscillations in the other groups having significantly smaller areas. Rise time (Figure 11C) was longer in the control groups relative to 30K. The rise time for Ca^2+^ oscillations in the presence of 30K with ryanodine was shorter than control in the normoxic but not hypoxic condition. Oscillatory durations (Figure 11D) showed a pattern similar to the AUC, with events in the 30K group having a shorter duration compared to that of controls. The duration following ryanodine treatment was similar to that of control conditions. The decay in the oscillation (Figure 11E) for the controls was longer in the hypoxic as compared to normoxic group. 30K treatment shortened the decay time in both groups, while decay times increased following ryanodine treatment, being similar in length to control conditions.

**FIGURE 12:**
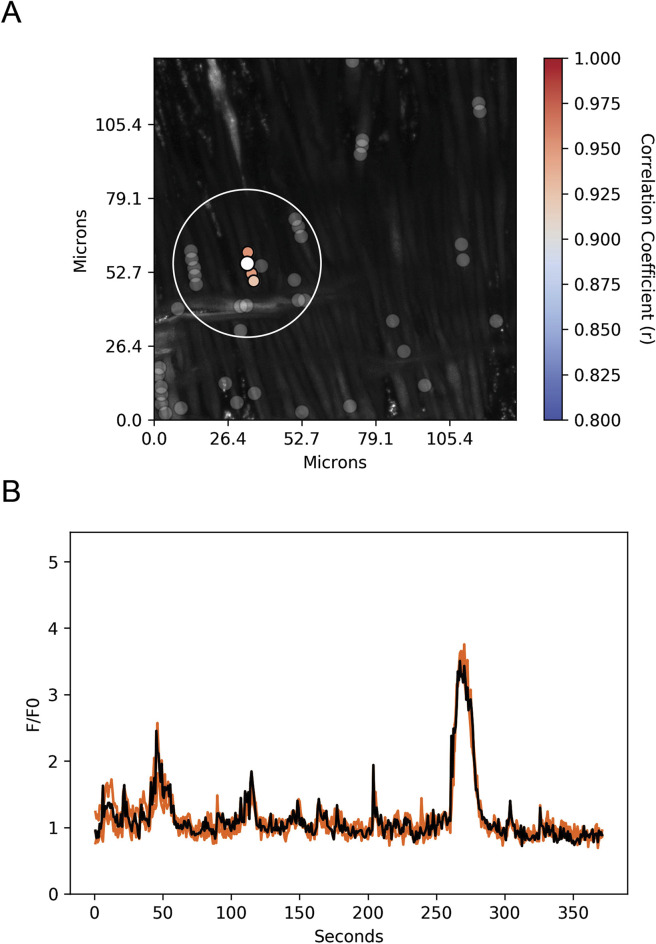
Spatial and temporal correlations of spontaneous Ca^2+^ oscillations in middle cerebral arterial myocytes from a normoxic fetal sheep recorded *en face* under control conditions. **(A)** Maximum intensity projection for Fluo-4 fluorescence of recorded cells using laser scanning confocal microscopy. Highly correlated temporal events are colored and plotted around the center (reference) region of interest (ROI, white dot). Gray dots show ROIs of spontaneous Ca^2+^ oscillations that had less than an 80% temporal correlation with the reference (white) ROI. The large white open circle shows a 26 μm diameter that was used to determine the spatial correlation measurements depicted in Figure 12. **(B)** Fluorescence intensity tracing showing spontaneous Ca^2+^ oscillations. The black line is the reference tracing, and the orange lines are the other individual ROIs, with correlation coefficients greater than 80% temporal correlation plotted surrounding the reference ROI (white dot in A). Key shows the degree of correlation of each ROI relative to the reference ROI. Recording was made with a 1.2 NA ×63 water immersion objective. Image brightness and contrast were adjusted to improve visualization of cells.

Spatial and temporal correlations between the ROI Ca^2+^ transients were then examined. Increases in the correlation coefficient for temporally aligned Ca^2+^ oscillations denoted greater levels of event “friendship.” Temporally related correlations between the Ca^2+^ events were then identified by those events that had a correlation coefficient of r ≥ 0.8 (Figure 12A) (Shen et al., 2018). Results from a representative recording are provided in Figure 12. Figure 12A shows a maximum intensity projection from a time lapse recording with overlaid regions of interest that have varied levels of correlations. Figure 12B shows the fluorescence over time (black line) for the center ROI along with the fluorescence traces for those regions of interest that were temporally correlated. The spatial relationship between events was then evaluated (Figure 12B). ROIs that were within a 100 pixel (∼26 µm) radius of a reference ROI were denoted “neighbors,” as delineated by the larger white circle on the image in Figure 12B (Shen et al., 2018; Reid et al., 2021).

**FIGURE 13:**
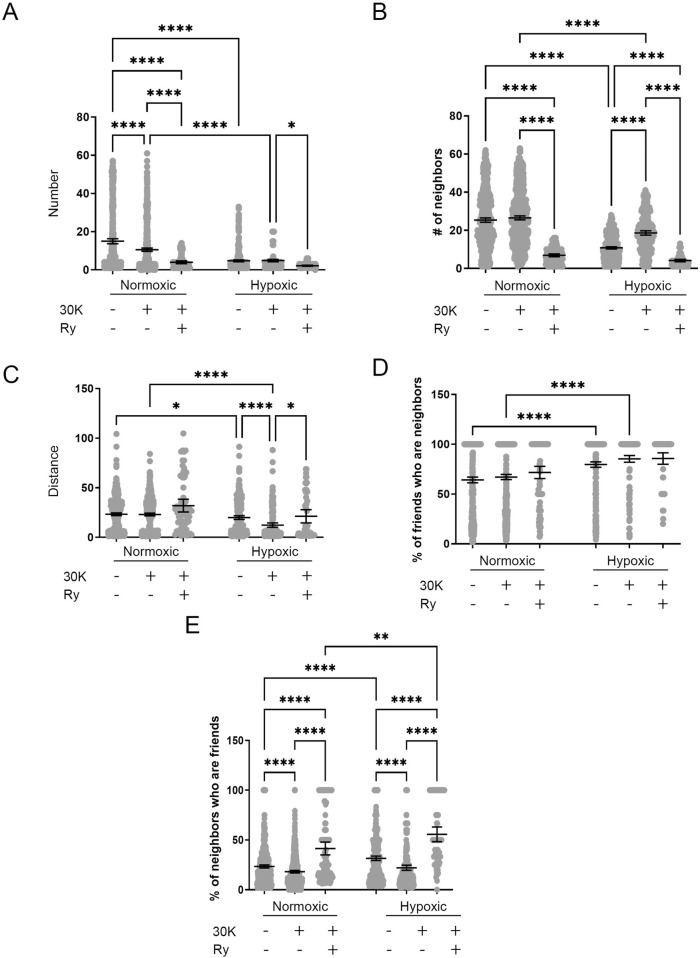
Long-term hypoxia impairs the influence of membrane depolarization on the friendship and neighborliness of Ca^2+^ oscillations in MCA myocytes of fetal sheep. **(A–E)** Arteries were analyzed for **(A)** number of correlated ROIs (friends), **(B)** number of nearby ROIs (neighbors), **(C)** distance between correlated ROIs, **(D)** percentage of correlated ROIs that were nearby, and **(E)** percentage of nearby ROIs that were correlated. Show are the individual data points for each ROI (gray dots) along with the mean ± 95% CI for control, 30K, and 30K + Ry as denoted on the graph. *P < 0.05, **P < 0.01, ***P < 0.001 indicates significance based on a Kruskal–Wallis ANOVA with a Dunn’s multiple comparisons test based on ranks. Numbers of observations for each recording condition and group are provided in Table 2.

LTH and membrane depolarization had diverse effects on the numbers of friends, with summary data provided in Figure 13A. LTH decreased the number of friends under control conditions and following membrane depolarization. Membrane depolarization decreased the number of friends in the normoxic group but not the hypoxic group. Ryanodine treatment reduced the number of friends independent of altitude. The number of neighbors was also affected by LTH and membrane depolarization. Figure 13B shows that like friendship, the number of neighbors, which are those events within a 26 micron radius of a reference event, was reduced with LTH under control and 30K conditions. The number of neighbors was unchanged following membrane depolarization in the normoxic group but was increased in hypoxic conditions, while ryanodine reduced the number of neighbors in both groups. Figure 13C delineates the distance between friends. LTH reduced the distance between friends in both control and depolarized conditions. Membrane depolarization reduced the distance between friends in hypoxic but not normoxic conditions. Ryanodine treatment increased the distance between friends in hypoxic but not normoxic conditions.

Figure 13D provides the percentage of friends who are neighbors, illustrating the spatial and temporal relationships between Ca^2+^ oscillations. Overall, a majority of temporally correlated oscillatory events (friends) are closely associated (neighbors). Notably, these spatially correlated neighbor events that were also temporally correlated typically arose within the same cell as opposed to adjacent cells. Long-term hypoxia resulted in an increase in the percentage of friends that were neighbors in both the control and 30K conditions. Figure 13E presents the percentage of neighbors who are friends, which is dependent on the temporal relationship between Ca^2+^ oscillatory events that are spatially related. Events that occur in nearby regions of interest are not necessarily temporally correlated, though they are influenced by LTH and membrane depolarization. LTH increased the percentage of neighbors that are friends for control, 30K, and ryanodine conditions. Membrane depolarization reduced the percentage of neighbors who are friends in myocytes regardless of their exposure to LTH, while ryanodine increased the percentage.

## 4 Discussion

The current study builds from our group’s work examining the impact of intrauterine long-term hypoxia on the regulation of vascular reactivity, Ca^2+^ signaling, and potassium channel activity in the fetal Cerebrovasculature (Lin et al., 2003; Tao et al., 2015; Silpanisong et al., 2017; Thorpe et al., 2017; Ducsay et al., 2018; Reid et al., 2021). The studies illustrate that fetal middle cerebral arterial myocytes have depolarization modulated ryanodine-receptor mediated Ca^2+^ spark activity and whole-cell Ca^2+^ oscillatory activity that are each modestly decreased following LTH. Secondarily, the data show that following LTH there is an increase in coupling efficiency between Ca^2+^ spark events and BK_Ca_ channel activity. The data suggest that the increased Ca^2+^ spark - BK_Ca_ coupling more than offsets any potential decreases in Ca^2+^ spark activity such that spontaneous transient outward currents are activated at more negative membrane potentials with activity being greater across a wide-range of physiologically relevant membrane potentials.

We found it interesting that fetal middle cerebral arterial myocytes have high Ca^2+^ spark activity regardless of whether they were depolarized or not. The high percentage of cells with Ca^2+^ sparks is reminiscent of what we observed in myocytes of pregnant sheep uterine arteries (Hu et al., 2019) and those from the mouse mesentery (Harraz et al., 2015). However, the finding is distinct from what we previously reported in adult rat middle cerebral (Hashad et al., 2017) as well as basilar and pulmonary arterial myocytes of fetal sheep (Hadley et al., 2012; Reid et al., 2021), each of which had far less activity than fetal MCA of the current study. The differences in Ca^2+^ spark activity may be due to differences in channel abundance or ryanodine receptor isoform expression (Smith et al., 1998; Cheng and Lederer, 2008). Differences in channel activity could also be mediated through complexities in the regulation of ryanodine receptors by various signaling pathways, potentially including differences in cell membrane potential, cytosolic Ca^2+^ levels, channel phosphorylation, channel oxidation, or myriad other regulatory processes (Cheng and Lederer, 2008; Gong et al., 2021; Ogawa et al., 2021).

We surmise that the physiological premise for the differences in Ca^2+^ spark activity in uterine, basilar, pulmonary, and mesenteric vessels may be because of the varying oxygenation and nutrient demands of the tissues, which have vastly different perfusion and concomitant vasodilatory needs. In the case of the uterine vasculature, pregnancy increased Ca^2+^ spark activity (Hu et al., 2019), which stimulates STOCs and vasodilation of uterine arteries, resulting in increased uterine blood flow and nutrient supply to the placenta, which in turn allows the fetus to thrive (Ducsay et al., 2018). Comparatively, pulmonary arterial myocytes in the fetus may be fairly quiescent because the fetal lung does not require much blood flow as the organ does not provide for gas exchange until after birth, when spark activity is increased (Papamatheakis et al., 2013; Ducsay et al., 2018). Conceivably, the differences in Ca^2+^ spark activity between basilar and middle cerebral arterial myocytes may be because these vessels perfuse different regions of the brain. Basilar arteries perfuse the brain stem, cerebellum, and occipital lobes. Middle cerebral arteries in comparison provide blood to most of the lateral surface of the cerebral hemispheres, the temporal lobes, and many deep regions of the brain. Before birth the brainstem may not need to modulate its nutrient supply to the same extent as the cerebral cortex. Since fetuses have diurnal patterns of activity, which would presumably coincide with varying levels of cortical activity (Bradford et al., 2019; Yao et al., 2024), nutrient requirements to the cerebral cortex would follow suit. The differences in activity between rat and sheep MCA suggest there are additional complexities in the regulation of the Ca^2+^ signals. Conceptually, the differences in the Ca^2+^ signals between sheep and rats may be the result of differences in brain size and related vascular blood flow due to the varied architecture of the two species, with the sheep brain being roughly 50 fold larger than that of a rat (Banstola and Reynolds, 2022). The developmental stage may also potentially be a factor in some of these differences. Fetal MCAs may have a relatively high coupling efficiency of Ca^2+^ sparks to STOC activity in order to maintain cerebral vasodilation and nutrient delivery to ensure proper fetal brain development with relatively low and constant blood pressure (Ducsay et al., 2018). Adult MCAs in comparison may have lower coupling efficiency between Ca^2+^ sparks and STOCs to maintain a greater degree of vasoconstriction and vasoregulatory range as well as mitigate the risk of hemorrhage as systemic blood pressure is significantly greater in the adult relative to the fetus.

The modest impact of membrane depolarization and ryanodine treatment on the amplitude, kinetic, and spatial aspects to Ca^2+^ sparks extend from our previously published studies. While we do not know if these modest changes translate into biological significance they are interesting. In the current studies, there was greater impact of membrane depolarization on spatial-temporal aspects to Ca^2+^ sparks in MCA myocytes as compared to our previously published work in basilar arterial myocytes (Reid et al., 2021). Such findings are not unfounded as we have previously observed age-related increases in Ca^2+^ spark amplitude and width in pulmonary arterial myocytes as a sheep grows from being a fetus, to a 2 week old newborn, and then to an adult (Hadley et al., 2012). We do not yet fully understand the mechanistic and physiological processes that govern how or why there are modifications to the spatial-temporal aspects to Ca^2+^ spark activity by membrane depolarization or animal age between pulmonary, basilar, and middle cerebral arterial myocytes. The age-related differences in Ca^2+^ spark activity in pulmonary arterial myocytes may be entwined with the physiology of the fetal as compared to the newborn and adult lung. Before birth, blood flow to the fetal lung is largely restricted because the lung is not used for respiration (Papamatheakis et al., 2013). The heightened need for gas exchange with birth prompts the vessels to dilate, which allows for increased alveolar blood flow and gas exchange. Accordingly, ryanodine receptor driven Ca^2+^ sparks may become more prominent so as to enhance vasodilation (Porter et al., 2001; Resnik et al., 2006). The modifications in spatial and temporal aspects to the Ca^2+^ sparks in the MCA may also reflect complex changes in ryanodine receptor expression and regulation, along with changes in sarcoplasmic reticulum Ca^2+^ handling (Leslie et al., 2021) and Ca^2+^ sequestration and extrusion mechanisms at the plasma membrane and other organelles including the mitochondria (Keizer and De Young, 1993; Keizer and Levine, 1996; Keizer and Smith, 1998; Smith et al., 1998).

We are intrigued by the finding that intrauterine long-term hypoxic stress caused a reduction in the number of MCA myocytes with Ca^2+^ sparks and the frequency of Ca^2+^ spark activity in cells that were depolarized. The impairment in Ca^2+^ spark activity following LTH is similar to pulmonary arterial myocytes in the fetus as well as adult basilar arterial myocytes and uterine arteries of pregnant animals in which Ca^2+^ spark activity is reduced by LTH (Hadley et al., 2012; Hu et al., 2019; 2020; Reid et al., 2021), but dissimilar to fetal basilar arteries where Ca^2+^ spark activity was preserved (Reid et al., 2021). From a functional perspective, the reduction in Ca^2+^ spark activity is likely to be coupled to dysregulation of tissue blood flow and important to varying vascular pathologies in the mother and fetus associated with intrauterine LTH (Ducsay et al., 2018; Jackson, 2021). The mechanistic underpinnings for the reduction in Ca^2+^ spark activity with LTH, however, remains an open area of investigation.

The differential effects of ryanodine treatment on Ca^2+^ spark characteristics in the normoxic and hypoxic groups are worth noting; where ryanodine in the presence of 30K decreased Ca^2+^ spark amplitude and duration in the normoxic group but increased the amplitude without any change in duration in the hypoxic group. Mechanistically these findings may be related to the actions of ryanodine. Ryanodine only binds when the channel is in the open state and can either lock the channel in a subconductance state or can block the channel (Du et al., 2001; Janiak et al., 2001; Cheng and Lederer, 2008). Because of the binding properties of ryanodine one possibility is that our findings with ryanodine are spurious and are dependent on heterogeneity in the cell populations we are examining. Still, ryanodine decreased Ca^2+^ spark firing rates by nearly 10-fold, illustrating that ryanodine receptor activity is reduced by the plant alkaloid. These differences in Ca^2+^ spark activity between normoxic and LTH myocytes following ryanodine treatment open the possibility that long-term hypoxia modifies channel gating behavior. Potentially this involves alterations in LTH-mediated regulation of ryanodine receptors through various channel phosphorylation and other signaling mechanisms or via changes in RyR isoform expression (Cheng and Lederer, 2008; Jackson, 2021; Song et al., 2021).

The Ca_v_ - RyR - BK_Ca_ channel signaling axis is of critical importance to feedback regulation of vascular tone and tissue blood flow. The adaptation of the signaling axis following LTH, such that BK_Ca_ channel activity is accentuated at more negative membrane potentials, on the surface, suggests that the arterial myocytes of hypoxic fetuses may be relatively relaxed compared to those of normoxic animals. However, the structural and functional changes to the fetal MCA following LTH that our group have previously published (Long et al., 2002; Adeoye et al., 2015; Tao et al., 2015; Silpanisong et al., 2017; Thorpe et al., 2017; Ducsay et al., 2018; Sorensen et al., 2021) provide a different perspective. LTH causes complex effects on arterial reactivity, with an overall loss in the vasoregulatory range through depression in vasodilation and vasoconstriction capacity (Longo et al., 1993; Silpanisong et al., 2017; Thorpe et al., 2017; Ducsay et al., 2018). This includes loss of a role for BK_Ca_ channels in feedback mediated dilation during norepinephrine or serotonin stimulation (Long et al., 2002; Thorpe et al., 2013). Previous evidence from our group also illustrates there is a pronounced loss in contractile smooth muscle through a phenotypic switch of the arterial myocytes towards a synthetic phenotype (Adeoye et al., 2015; Silpanisong et al., 2017; Thorpe et al., 2017; Sorensen et al., 2021). Some of these alterations are linked to changes in G-protein coupled receptor mediated responses (Longo and Pearce, 1998; Adeoye et al., 2015; Silpanisong et al., 2017; Ducsay et al., 2018; Pearce, 2018). These modifications in G-protein coupled receptor activity and their related signaling pathways are likely to impact the regulation of BK_Ca_ channel activity (Tykocki et al., 2017; Peixoto-Neves and Jaggar, 2023). From a functional perspective, the augmented BK_Ca_ channel activity observed in our current studies may act as a compensatory response to maintain blood flow in response to dysregulation in arterial structure and function (Tykocki et al., 2017; Ducsay et al., 2018).

The enhancement in BK_Ca_ channel currents following LTH in fetal MCA parallels work performed by our group in basilar arteries from fetal animals (Tao et al., 2015) and uterine arteries from pregnant sheep (Hu et al., 2019). The increase in membrane current amplitude following LTH could be explained by either an increase in channel expression or channel open probability. Previously published Western blot data from our group indicates that BK_Ca_ alpha subunit expression is reduced in fetal MCA following LTH (Long et al., 2002; Thorpe et al., 2017). While it is possible that total BK_Ca_ alpha subunit expression does not reflect cell surface expression this seems unlikely based on previous studies showing that a high percentage of subunits are inserted in the plasma membrane (Peixoto-Neves and Jaggar, 2023). Instead, we conjecture that the increase in STOC frequency and amplitude are the result of a change in channel open probability as our previous data has shown LTH results in an increase in open dwell times of single channel currents in fetal basilar myocytes (Tao et al., 2015).

The present studies were not designed to directly assess the importance of BK_Ca_ channels to vasoregulation, the role of Ca^2+^ sparks in that process, or in feedback modulation of arterial reactivity in response to changes in arterial pressure or agonist stimulation. The distinctive difference in the findings of the current studies, and especially the voltage dependence of activation for BK_Ca_ currents in fetal normoxic myocytes compared to our previous work examining arterial reactivity strongly supports the need to perform additional studies. These experiments would focus on resolving the cellular and molecular mechanisms and functional consequences associated with alteration in the Ca_v_-RyR- BK_Ca_ signaling axis in the fetal MCA following intrauterine LTH and the activation of BK_Ca_ channels in response to increases in vascular pressure as compared to agonist stimulation (Knot and Nelson, 1995; Nelson et al., 1995; Long et al., 2002; Thorpe et al., 2013).

The large negative shift in the voltage dependence of activation of STOC activity following LTH, such that the BK_Ca_ channels are activated at more hyperpolarized membrane potentials provide additional insights and further illustrate there are mechanistic changes in the gating behavior of the channels. These findings are reminiscent of previous work on BK_Ca_ channel function from our group performed in fetal basilar arteries where LTH elicited multiple changes in BK_Ca_ channel function (Tao et al., 2015). Following LTH, BK_Ca_ channels had an increase in the Ca^2+^ affinity of the channel, modification in the responses to channel phosphorylation, and facilitation of channel activation at more negative membrane potentials, as well as increased cell surface expression of the BK_Ca_ beta subunit (Tao et al., 2015). We do not know all the effects of LTH on BK_Ca_ channels in fetal sheep MCA and whether the changes parallel the effects we have reported in the basilar arterial myocytes (Tao et al., 2015). Conceivably hypoxia could cause similar changes in the ability of Ca^2+^ or channel phosphorylation to regulate channel activity, which may coincide with genetic or epigenetic changes in the BK_Ca_-alpha subunit that affect channel regulation (Lin et al., 2003; Kyle and Braun, 2014; Tao et al., 2015; Dopico et al., 2018). The differences in voltage dependence of activation and BK_Ca_ channel activity may also arise from changes in the expression and association of other regulatory players including lipid regulation or other post translational modification through oxidation, nitrosylation, or ubiquitination of the channel (Dopico and Bukiya, 2014; Kyle and Braun, 2014; Dopico et al., 2018). Another possibility is that LTH increases surface expression of beta or gamma subunits and interaction with the BK_Ca_ alpha subunits (Gonzalez-Perez and Lingle, 2019), although our published Western blot studies indicate there is not an increase in beta subunit expression in fetal MCA (Thorpe et al., 2017). Importantly, the stoichiometry between beta and alpha subunits alters the voltage dependence of activation and Ca^2+^ sensitivity of the channel. Increasing the proportion of beta- to alpha-subunits is well known to induce a leftward shift in the voltage dependence for activation (Cox and Aldrich, 2000). The cell surface expression of beta subunits is dependent not only on subunit expression but also on translocation to the plasma membrane (Peixoto-Neves and Jaggar, 2023). Indeed, previous evidence shows LTH increases the plasma membrane expression of beta-subunits in fetal basilar arterial myocytes (Tao et al., 2015), and pregnancy increases their expression in uterine arteries (Song et al., 2021). Translocation of BK_Ca_ beta subunits to the plasma membrane and interaction with BK_Ca_ alpha is a dynamically regulated process (Leo et al., 2014), which can be negatively impacted by hypertension, diabetes, and other vascular diseases (Yang et al., 2013; Nieves-Cintrón et al., 2017; Dopico et al., 2018; Peixoto-Neves and Jaggar, 2023). Assessing the impact of LTH on changes in regulatory subunits, accessory proteins, regulatory signaling processes, as well as channel translocation and their impacts on vasoregulation are important and open areas of inquiry.

The LTH-related impairments in the amplitude and spatial-temporal aspects to Ca^2+^ oscillatory activity in myocytes of fetal sheep MCA under control conditions and with membrane depolarization is noteworthy. This finding builds from previous work performed in LTH fetal sheep illustrating a marked reduction in vascular contraction in middle cerebral arteries following gestational LTH (Longo et al., 1993; Ducsay et al., 2018). However, the reduction in the magnitude of the Ca^2+^ oscillations following LTH in the MCA differs from our recent work in basilar arteries from fetal sheep where the spatial-temporal aspects to the Ca^2+^ signals were largely preserved following LTH. Regional heterogeneity in responsiveness builds from previous studies showing differences in fetal perfusion of cortex versus brainstem during hypoxia (Ashwal et al., 1984) and further supports the general premise that arterial function depends on the tissue demands. The differences we note regarding the impact of LTH on basilar and MCA arterial systems with regards to the regulation of blood flow as well as Ca^2+^ signaling speak to the need for studies to be performed in multiple arterial systems and to limit overgeneralization of findings from one vessel bed to another.

The lack of coupling between the oscillatory Ca^2+^ signals among MCA myocytes was expected. We have previously published that myocytes in pulmonary and basilar arteries oscillate independently from one another (Shen et al., 2018; Reid et al., 2021) and that the level of cell interaction is unaffected by tissue hypoxia or membrane depolarization. The lack of spatial or temporal coordination in Ca^2+^ signaling between vascular myocytes also supports previous evidence that there is limited cell-to-cell communication via gap junctions (Mironova et al., 2024).

## 5 Perspective

The Ca_v_-RyR-BK_Ca_ signaling axis is crucial to the regulation of vascular tone and blood flow to the brain. Feedback regulation of membrane potential allows cerebral arteries to finely tune their diameter in response to various physiological signals, ensuring a regulated supply of oxygen and nutrients to brain tissues. The LTH mediated adaptation in coupling between membrane potential, Ca^2+^ sparks, and BK_Ca_ channels in fetal middle cerebral arterial myocytes shown in the current work sheds additional light on known disturbances in vascular tone due to LTH prenatal stress; dysfunction that may lead to hyperperfusion and increased risk of intracerebral hemorrhage or other related cerebrovascular pathologies. Fetal hypoxia increases cerebral blood flow due to cerebral vascular dilation, favoring and maintaining fetal brain development in the presence of hypoxia. This is supported by our data showing an increase in MCA STOCs in the fetal brain by LTH. Cerebral vasodilation and an increase in cerebral blood flow in the fetus are likely to have a protective mechanism for brain development *in utero* under hypoxia. The dilation may, however, cause a problem after birth when blood pressure increases, elevating the risk of hemorrhage in the newborn brain. Fully understanding the functional consequences and molecular underpinnings of these changes, however, will require further interrogation.

## Data Availability

The raw data supporting the conclusions of this article will be made available by the authors, without undue reservation.
